# Emerging Roles of Gut Microbial Modulation of Bile Acid Composition in the Etiology of Cardiovascular Diseases

**DOI:** 10.3390/nu15081850

**Published:** 2023-04-12

**Authors:** Tess Yntema, Debby P. Y. Koonen, Folkert Kuipers

**Affiliations:** 1 Department of Pediatrics, University of Groningen, University Medical Center Groningen, 9713 GZ Groningen, The Netherlands; t.yntema@umcg.nl (T.Y.); d.p.y.koonen@umcg.nl (D.P.Y.K.); 2 European Research Institute for the Biology of Ageing (ERIBA), University of Groningen, University Medical Center Groningen, 9713 GZ Groningen, The Netherlands

**Keywords:** gut microbiota, bile acids, cardiovascular disease, atherosclerosis

## Abstract

Despite advances in preventive measures and treatment options, cardiovascular disease (CVD) remains the number one cause of death globally. Recent research has challenged the traditional risk factor profile and highlights the potential contribution of non-traditional factors in CVD, such as the gut microbiota and its metabolites. Disturbances in the gut microbiota have been repeatedly associated with CVD, including atherosclerosis and hypertension. Mechanistic studies support a causal role of microbiota-derived metabolites in disease development, such as short-chain fatty acids, trimethylamine-N-oxide, and bile acids, with the latter being elaborately discussed in this review. Bile acids represent a class of cholesterol derivatives that is essential for intestinal absorption of lipids and fat-soluble vitamins, plays an important role in cholesterol turnover and, as more recently discovered, acts as a group of signaling molecules that exerts hormonal functions throughout the body. Studies have shown mediating roles of bile acids in the control of lipid metabolism, immunity, and heart function. Consequently, a picture has emerged of bile acids acting as integrators and modulators of cardiometabolic pathways, highlighting their potential as therapeutic targets in CVD. In this review, we provide an overview of alterations in the gut microbiota and bile acid metabolism found in CVD patients, describe the molecular mechanisms through which bile acids may modulate CVD risk, and discuss potential bile-acid-based treatment strategies in relation to CVD.

## 1. Introduction

Cardiovascular diseases (CVD) represent the number one cause of death globally, taking approximately 17.9 million lives each year, i.e., 32% of all deaths in 2019 [1]. Although CVD mainly affects the elderly, its incidence in younger people is increasing [2]. In accordance with the continuous ageing of the global population, CVD prevalence is steadily rising [3,4]. Atherosclerosis, a chronic low-grade inflammatory disorder of the vascular wall, is the main pathophysiological condition to result in CVD [5]. Atherosclerosis is characterized by the build-up of cholesterol-engulfed macrophages and other inflammatory immune cells in the vascular wall [2], causing coronary artery disease (CAD), ischemic stroke, or peripheral artery disease, through luminal narrowing of the blood vessels, plaque rupture, and thrombus formation [6].

Traditional risk factors of atherosclerosis include hyperlipidemia, hypertension, and chronic inflammation [7]. Although the scientific community has made tremendous progress in understanding, preventing, and modulating these risk factors, a significant residual cardiovascular risk remains, highlighting the urgent need for additional treatment strategies [8]. In recent years, non-traditional drivers of atherosclerosis, such as the gut microbiota, have gained attention [2,9]. An imbalanced gut microbiota, or dysbiosis, has been associated with cardiometabolic diseases, i.e., atherosclerosis, obesity, type 2 diabetes (T2D), and non-alcoholic steatohepatitis (NASH) [10,11]. Dysbiosis can lead to increased intestinal permeability, which results in the translocation of the bacteria and endotoxins that promote chronic inflammation [12]. In addition, the gut microbiota has been implicated in CVD through the actions of microbiota-derived metabolites, such as short-chain fatty acids (SCFAs) [13], trimethylamine (TMA) [14], and bile acids (BAs) [15]; the latter will be elaborated on in this review.

BAs comprise a class of cholesterol metabolites, whose hepatic synthesis and eventual fecal excretion constitute the major pathway of cholesterol removal from the human body. Over 40 years ago, BA sequestrants (BAS) were among the first FDA approved drugs to lower cholesterol levels in CVD [16]. BAS bind to BAs and prevent their reabsorption from the intestine to promote their fecal loss. To compensate those that are lost, the liver produces more BAs from cholesterol, leading to upregulation of the hepatic low-density lipoprotein (LDL) receptor expression and lowering of the plasma LDL levels. Other than the lipid-lowering effects, clinical studies have shown an increase in coronary lumen diameter and lower cardiovascular risk upon BAS administration in CAD patients [17,18,19]. BAs function physiologically as detergents of lipids and fat-soluble vitamins to facilitate their intestinal absorption. In the last decennia, BAs were also shown to exert endocrine functions within and outside the enterohepatic circulation through the activation of a variety of BA receptors (BAR). Upon BAR activation, BAs can mediate lipids, as well as glucose metabolism and inflammation [20]. Interestingly, the expression of several BAR in cardiovascular tissues, such as the vasculature and immune system, highlights the potential of BA modulation in CVD [21]. In this review, we aim to provide an overview of (microbiota-modulated) BA metabolism and describe their potential biological impact thereof, especially in relation to CVD risk. Finally, we will discuss novel BA-based therapeutic targets that could be useful in the management of CVD.

## 2. Bile Acids Are Synthesized by the Liver and Extensively Metabolized by the Microbiota

**Figure 1:**
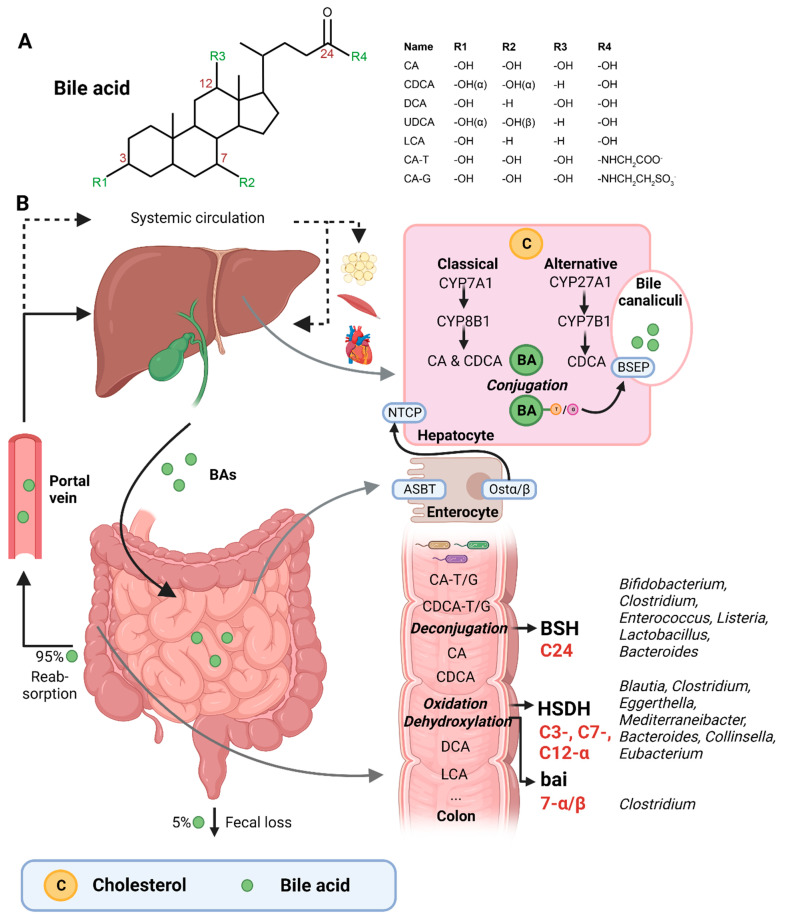
(**A**) Structural formula of bile acid. (**B**) Hepatic BA synthesis, enterohepatic circulation, and microbial BA modifications in the human body. BA = bile acid; CA = cholic acid; CDCA = chenodeoxycholic acid; DCA = deoxycholic acid; UDCA = ursodeoxycholic acid; LCA = lithocholic acid; TCA = taurocholic acid; GCA = glycocholic acid; C = cholesterol; BSEP = bile salt-export pump; NTCP = Na^+^-taurocholic acid co-transporting polypeptide; ASBT = apical sodium-dependent BA transporter; Ostα/β = organic solute transporter α/β; BSH = bile salt hydrolases; HSDH = hydroxysteroid dehydrogenase; bai = BA inducible genes. This illustration was created with Biorender.com (accessed on 1 February 2023).

BAs are amphipathic cholesterol derivates, characterized by one or more hydroxyl groups at their steroid nucleus (in mammals, commonly at positions 3, 7, and/or 12) and a shortened side chain bearing a carboxylic acid group [22] (Figure 1A). BAs are exclusively synthesized in hepatocytes in a process that involves a cascade of enzymatic steps in different compartments of the cell [23,24,25]. The products of this multi-step process are the so-called primary BAs, i.e., cholic acid (CA) and chenodeoxycholic acid (CDCA) in humans, which are products of the well-established “classical” or “alternative” pathways [26].

The first step of the classical pathway is catalyzed by the rate-controlling enzyme cytochrome P450 cholesterol 7α-hydroxylase (CYP7A1), which yields 7α-hydroxycholesterol [23,27] (Figure 1B). The alternative pathway is initiated by sterol 27α-hydroxylase (CYP27A1), and further processed by oxysterol 7α-hydrolase (CYP7B1) [27]. The 7α-hydroxylated intermediates from both the classical and alternative pathway undergo several sterol ring modifications, as well as side chain oxidation and shortening, leading to the production of CDCA [26,27]. In addition, the classical pathway also generates CA, which involves the activity of sterol 12α-hydroxylase (CYP8B1) [28]. Of note, rodents produce additional forms of primary BAs in the liver—α- and β muricholic acids (MCAs) and ursodeoxycholic acid (UDCA)—which are derived from CDCA by mouse/rat-specific cytochrome P450 2C70 (Cyp2c70) [26,29].

Primary BAs are conjugated with either taurine or glycine at the C24 position, which allows for their active secretion into the bile and decreases their pKa, and hence prevents passive reabsorption in the upper intestine [25]. BAs are secreted into the bile canaliculi by the bile salt-export pump (BSEP) and stored in the gallbladder in high mM range [26,30] (Figure 1B).

Upon meal intake, cephalic-phase-induced cholecystokinin secretion from the duodenum stimulates gallbladder contraction, leading to the discharge of stored BAs into the intestine [25]. In the intestinal lumen, BAs solubilize lipids, including cholesterol, and fat-soluble vitamins. Most primary BAs are actively reabsorbed in the ileum by the apical sodium-dependent BA transporter (ASBT) [25]. A small proportion of BAs escape uptake and reach the colon, where the gut microbiota can convert primary into secondary BAs (lithocholic acid (LCA) and deoxycholic acid (DCA)), a process that involves three major groups of bacterial enzymes (discussed in the subsequent section) [24]. Unconjugated secondary BAs are relatively hydrophobic and can thus be passively reabsorbed from the colon [20]. Most BAs (95%) are reabsorbed in the ileum or colon, and via the portal vein they are delivered back to the liver, whereafter they are taken up by Na^+^-taurocholic acid co-transporting polypeptide (NTCP), in order to finalize a process called the enterohepatic circulation. In the liver, unconjugated primary and secondary BAs are re-conjugated and secreted into bile. Only a minor amount (~5%) of BAs, which are not taken up in the ileum or colon, are excreted in feces per cycle [20]. This loss of BAs is compensated by the de novo synthesis of BAs in the liver to maintain the size of the circulating BA pool [20]. 

A relatively small fraction of BAs escape first pass clearance by the liver and flow into the systemic circulation, with concentrations in the µM range [30]. From the peripheral circulation, BAs can reach multiple organs, including adipose tissue, muscle, and the heart [31,32]. BAs exert hormone-like functions by acting as signaling molecules and activate several BARs, i.e., nuclear receptors—farnesoid X receptor (FXR), vitamin D receptor (VDR), pregnane R receptor (PXR)—and membrane-bound receptors—Takeda G protein-coupled receptor (TGR5) and muscarinic receptors (MRs). Through these actions, BAs regulate their own homeostasis, as well as lipid and glucose metabolism, intestinal barrier function, cardiovascular functions, and inflammation [21,31,33]. Importantly, different BA species have dissimilar affinities for the activation of BARs (FXR: CDCA > LCA = DCA > CA; TGR5: LCA > DCA > CDCA > CA; VDR: LCA; PXR: LCA = CDCA = DCA = CA) [30,34]. Differences in BA pool composition, which are prominent across individuals [35], will thus affect BA signaling in a personalized manner.

## 3. Bacteria Involved in Bile Acid Metabolism

The three major groups of bacterial enzymes—bile salt hydrolases (BSHs), hydroxysteroid dehydrogenases (HSDHs), and bile-acid-inducible (bai) genes—are responsible for the generation of secondary BAs in the colon, leading to a major increase of the diversity of the BA pool. The major structural modifications include deconjugation, which is an obligatory first step; oxidation of hydroxy groups at the C3, C7, and C12 position; and 7α/β-dehydroxylation [24,25]. In addition, (7α/β, 3α/β) isomerization- and (5-H α/β) epimerization modifications give rise to UDCA and iso- and allo-BAs, respectively [26].

BSHs catalyze the deconjugation of the *N*-acylamide bond between primary BAs and taurine or glycine at the C24 position [25]. BSHs have been identified in several microbial genera, including *Bifidobacterium* [30,36], *Clostridium* [30,37], *Enterococcus* [30], *Listeria* [30,38,39], *Lactobacillus* [30,40,41], and *Bacteroides* [42]. Recently, computational analyses have shown that the human gut microbiota contains 591 intestinal bacterial strains within 117 genera with BSHs sequences [43]. BSHs, encompass seven [44] or eight sub-groups [43], showing differences in deconjugation ability. BSH-T3 shows the highest enzyme and deconjugation activity and is only found in *Lactobacillus* [43]. Recently, research has shown that, after deconjugation, the gut bacteria can also mediate the conjugation of the CA backbone with the amino acids phenylalanine, tyrosine, or leucine [45]. The microbial enzyme responsible for these BA modifications remains unknown. Interestingly, these amino acid BA conjugates are found in humans and are enriched in patients with inflammatory bowel disease or cystic fibrosis [45].

The second major group of bacterial enzymes are HSDHs, which oxidize and epimerize C3, C7, and C12 hydroxy groups of BAs. HSDH enzymes have been identified in the microbial genera *Blautia* (3α), *Clostridium* (3-, 7-, 12α), *Eggerthella* (3-, 12α), *Mediterraneibacter* (3α), *Bacteroides* (7α), *Collinsella* (7α), and *Eubacterium* (7α) [30]. Epimerization of hydroxy groups leads to a reversible change from the α to the β configuration, generating stable oxo-BAs as intermediates [25]. The reaction depends, in part, on the redox potential of the environment. For example, oxo-BAs are more present at the mucosal surface, where the redox potential is high, whereas less oxo-BAs are present in the lumen of the large intestine, where the redox potential is low [24]. Interestingly, the production of 12-oxoCDCA may reduce the formation of secondary BA DCA [46], which has been implicated in liver [47] and colon cancer [48], cholesterol gallstone formation [49], and CVD [50].

Bacteria that carry the bai operon produce enzymes that carry out 7-α/β dihydroxylation, resulting in the major secondary BAs DCA and LCA. Surprisingly, this metabolic pathway is only found in 0.0001% of colonic gut microbiota, belonging to the genera *Clostridium* [24,25,51]. Moreover, 7-α/β dehydroxylation only takes place after deconjugation, implicating a functional interplay between deconjugation and dehydroxylation [24].

Thus, the gut microbiota is responsible for diversifying the BA pool. This strongly affects BA signaling, as BAs have different affinities towards BARs [20]. Importantly, the regulation between the gut microbiota and BAs is reciprocal, meaning that BAs can also modulate the gut microbiota either by direct or indirect effects. For example, BAs can disrupt bacterial membranes or bind to intestinal FXR, promoting the expression of antimicrobial agents [52]. Moreover, conjugated BAs play an important role in the prevention of bacterial overgrowth in the proximal small intestine, which is relatively devoid of microbes under normal conditions [53]. Studies have shown that replenishing intestinal BA concentrations in BA-deficient rats abolished bacterial overgrowth in the small intestine [53]. On the other hand, bacteria that inhabit the intestinal tract must have specific resistance mechanisms to protect themselves against bile [54]. For example, *Lactobacillus* and *Bifidobacterium* produce proteins that are devoted to the efflux of BAs [54].

Of note, gut bacteria can also directly metabolize cholesterol in the intestine via dehydrogenase activity encoded by intestinal sterol metabolism A (*ismA*) genes, producing cholestanone and coprostanol [55]. These genes are found in the human gut microbiota in geographically diverse human cohorts and show a negative correlation with circulating cholesterol levels [55]. In addition, recent studies highlight the bacterial potential to sulfonate cholesterol and related steroids in the gut. The sulfotransferase enzyme is identified in the microbial species *Bacteroides thetaiotaomicron* [56,57]. The new (direct) lipid-metabolizing functions of the gut microbiota, which was originally thought to be performed by host enzymes, represent a great breakthrough in the understanding of cholesterol homeostasis.

## 4. Gut Microbiota Signatures in Cardiovascular Disease

Since the late 1980s, researchers have implicated a role of bacteria in atherogenesis [58]. In these early studies, the bacterium *Chlamydia pneumoniae* was associated with CAD and myocardial infarction. A few years later, bacterial DNA from many other bacteria genera/species were found in human atherosclerotic plaques [59,60,61], whereas healthy tissue (e.g., non-transplanted hearts) does not contain bacterial DNA [62]. Bacterial DNA has also been linked to inflammation, as the amount of bacterial DNA was found to correlate with the number of leukocytes in the plaque [59]. This suggests that the underlying pathophysiology of atherosclerosis may involve bacterial activation of the immune system. In addition, bacteria originating from the gut and the oral cavity matched with bacterial DNA present in atherosclerotic plaques and correlated with disease biomarkers [59]. Thus, the re-allocation of bacteria from the intestinal tract to the heart may contribute to disease development, which has sparked interest to evaluate the role of the gut microbiota in CVD.

Multiple cross-sectional studies have assessed the association of the gut microbiota with atherosclerosis (Table 1) [63,64,65,66,67,68,69,70,71] and hypertension (extensively reviewed in [72]). Despite differences in sequencing methods (16S versus shotgun) and downstream analyses, five out of nine studies showed a reduction in the genera *Roseburia* and *Faecalibacterium* in atherosclerosis patients [64,65,66,67,69]. These genera produce SCFAs, especially butyrate, which affect colonic motility and immunity, among other functions [73,74]. In addition, higher abundances of Gram-negative bacteria, such as *Enterobacter* [66,67], *Klebsiella,* and *Veillonella* [69], as well as *Streptococcus,* were found in atherosclerosis patients [67,70]. Gram-negative bacteria have lipopolysaccharides (LPS) on their outer membrane, which are pro-inflammatory. *Veillonella* and *Streptococcus* genera have been found in most human atherosclerotic plaques [59], and have both been implicated as pathogens [75,76,77,78].

Specifically looking at the major groups of bacterial enzymes, studies classified bacteria containing 3α-HSDH, 7α-HSDH, and/or BSH genes (indicated by [30,43]) in the gut microbiota of atherosclerosis patients, regardless of the direction of change (increased or decreased) (Table 1). For example, Toya et al. observed a higher abundance of *Ruminococcus gnavus* in atherosclerosis patients [68]. This species expresses 3α-HSDH enzymes and is an important producer of iso-DCA [79]. Moreover, the genera *Collinsella*, *Eubacterium,* and *Bacteroides* carry both 7α-HSDH and BSH genes, whereas *Roseburia*, *Streptococcus*, *Enterococcus,* and *Clostridium* only carry BSH genes (Table 1). Interestingly, BSH phylotypes are related to CVD [43,44]. For example, Karlsson et al. observed an increased abundance of *Collinsella* in the gut microbiota of atherosclerosis patients [64]. This genus carries BSH-T4 genes, whose relative abundance is significantly higher in atherosclerosis populations [43]. Moreover, multiple studies observed a reduced abundance of *Bacteroides* in the gut microbiota of atherosclerosis patients [66,71]. This genus carries BSH-T5/BSH-T6 genes, whose relative abundance are significantly lower in atherosclerosis populations [43]. This suggests that changes in BA-modifying bacteria in the gut microbiota may play a role in the pathophysiology of atherosclerosis.

Regarding the microbial alpha diversity, reflecting the number of bacterial species, the results are inconsistent across the studies enlisted in Table 1. In general, gut microbiota diversity has been found to negatively correlate with risk factors of atherosclerosis, such as obesity, hyperinsulinemia, hypertension, and dyslipidemia [72,80,81]. However, others have demonstrated that alpha diversity was either not different or increased in atherosclerosis patients compared with the controls [63,66,67,69,71]. These discrepancies could be due to limitations in the respective studies, such as omitting important confounders in their analyses (e.g., age, sex, and BMI) or assessing diversity with an estimation-based method (Chao1) [63]. Moreover, the temporal dynamics and intra- and interindividual heterogeneity of the gut microbiota underscores the difficulty in studying and comparing cross-sectional studies. Rodent models (especially germ-free models), which are more experimentally controlled, represent an invaluable tool for studying microbe–host interactions in the context of CVD.

In recent years, several studies have shown causal effects of the gut microbiota in the pathology of CVD [82,83,84,85]. For example, we recently showed that atherosclerosis was increased by 30% in (atherosclerosis-prone) low-density lipoprotein receptor knockout (*Ldlr^−/−^*) mice following transplantation with a pro-inflammatory gut microbiota and feeding a high-fat, cholesterol-enriched diet [85]. In addition, an in vitro screening protocol identified specific peptides that selectively modified bacterial growth [83]. These peptides can selectively partition into bacterial membranes, interrupt the transmembrane potential, and impair cell growth. Interestingly, Western-type diet-associated gut microbiota dysbiosis was remodeled by cyclic d,L-α-peptides towards the chow-diet microbial state, which was accompanied with rebalanced BA and SCFA homeostasis, suppressed production of pro-inflammatory cytokines and improved gut barrier integrity in *Ldlr^−/−^* mice [83]. Liu et al. transplanted germ-free mice with feces from CAD patients or healthy controls [84]. CAD patients were characterized by an altered gut microbiota composition and elevated serum levels of secondary BAs. Mice colonized with CAD microbiota showed an imbalanced BA composition with increased secondary BAs, worsened gut barrier permeability, and vascular dysfunction. In another study, supplementation with glycoursodeoxycholic acid (GUDCA) was shown to partly normalize Western diet-associated gut microbiota dysbiosis, which improved cholesterol homeostasis and local chronic inflammation and protected against atherosclerosis progression in (atherosclerosis-prone) apolipoprotein E-deficient (*ApoE^−^*^/*−*^) mice [82]. Changes in bacteria genera *Alloprevotella* and *Parabacteroides* positively, and *Turicibacter* and *Alistipes* negatively, were modulated by GUDCA and correlated with the plaque area in mice aortas [82]. Combined, these studies provide causal evidence that there is a functional interplay between gut bacteria and BAs, which affects CVD in mice.

## 5. Altered Bile Acid Metabolism in Cardiovascular Disease

Examining the composition of the gut microbiota at one single point in time ignores the complex nature of the gut microbiota as an ecosystem. Investigating functional shifts of the gut microbiota, including changes in gut-derived metabolites, helps to identify measurable read-outs of bacterial functions in health and disease. BAs might be a relevant gut metabolite in relation to CVD, despite controversy across studies [86,87,88,89]. Lower plasma BA levels have been reported in CVD [86,87]. Low serum BAs appeared to be independently associated with the presence and severity of CAD, especially for the presence of myocardial infarction (MI) [87]. These results were largely confirmed by Nguyen et al., although serum BA concentrations in both the control and CAD patients were lower compared with the previous study [86]. The latter study also observed doubled serum BAs in patients receiving statin therapy, suggesting that serum BAs levels are amendable by statin administration in CAD patients. Statins are a commonly described drug to lower cholesterol via the inhibition of β-Hydroxy β-methylglutaryl-CoA (HMG-CoA) reductase, the rate limiting enzyme in the cholesterol biosynthesis pathway [90]. Moreover, glyco-CDCA was two-fold higher in CAD patients [86] and, together with the total serum BAs, were predictors of CAD [86,87]. In contrast, a previous study comparing CAD and non-CAD patients did not demonstrate a significant association between serum BAs and CAD [88]. In addition, the total serum BAs are known to increase in patients with liver cirrhosis, which is associated with cardiac dysfunction [89]; total serum BAs are elevated up to 100 times the normal values in patients with cirrhotic cardiomyopathy [89], i.e., much more compared with CAD patients. Although inconsistency is found between studies, it appears that either low or extreme high serum BAs can be associated with CVD.

For zooming in on small-molecule metabolites in relation to CVD, untargeted metabolomics is a powerful tool to discover novel metabolites. Zhang et al. discovered that six metabolites were significantly altered in CAD patients [91]. LCA, together with 4-pyridocix acid and phosphatidylglycerol (20:3/2:0), showed the strongest positive correlation with CAD, defined as >80% stenosis in at least one artery. Of note, Chen et al. observed large inter-individual variability in plasma BA profiles in human obesity [35]. This variability suggests a more personalized approach to finding biomarkers and future therapeutic applications of BAs in CVD, although participants with recent cardiovascular events were excluded from this study [35]. Nevertheless, secondary BAs, i.e., DCA and LCA, were associated with diabetes and liver fat content, which are two risk factors of CVD, in these obese subjects.

In addition to plasma BAs, fecal bile acid excretion (BAE), which equals hepatic BA synthesis under steady state conditions, has also been associated with CAD [92,93,94]. CAD-patients were found to excrete less BAs, particularly DCA and LCA, compared with non-CAD controls [92,93,94]. A historical follow-up of 20 years showed that BAE was a significant independent parameter that predicted CAD in humans, in which BAE < 415 mg/day was associated with a higher long-term mortality due to CAD [94]. More specifically, 75% of the patients with BAE < 262.4/day developed a stroke relative to none of the patients with BAE > 622 mg/day [93]. BAE can thus serve as an interesting biomarker of CAD.

Thus, these studies provide evidence that measuring plasma and fecal BAs may aid in the assessment of the gut microbiota contributions to CVD. Understanding determinants of BA pool/metabolism and its reflection in CVD is important to rationalize their use as potential biomarkers and therapeutic targets.

## 6. Bile Acids as Mediators of Cardiovascular Disease Risk

**Figure 2:**
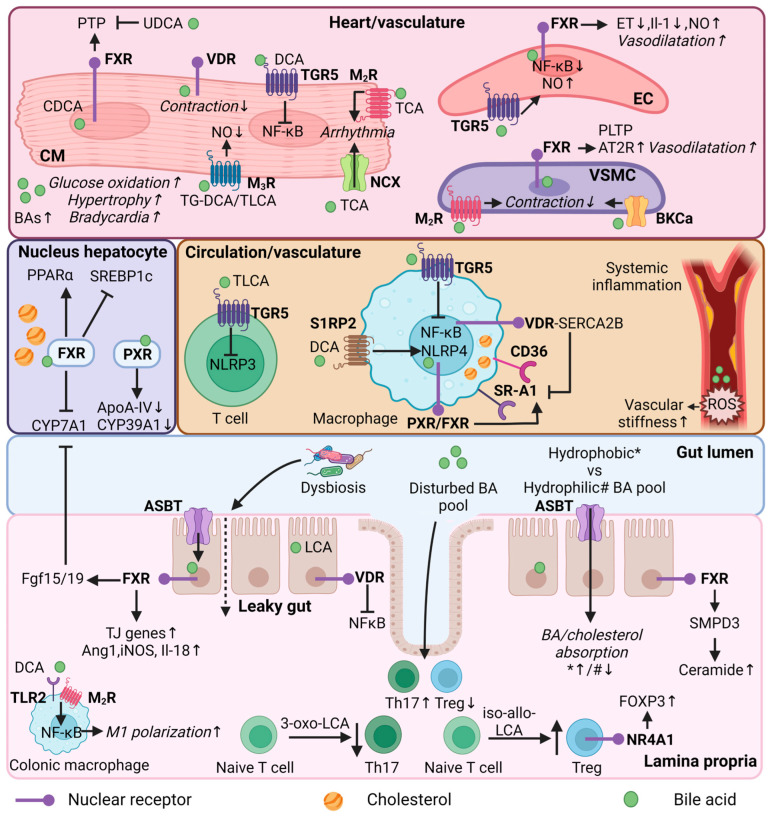
Bile acid regulation in lipid and immune metabolism, and heart function. Mechanistic effects of BAs in different organs (heart/vasculature/circulation/colon/lamina propria) or cell types (cardiomyocyte, endothelial cell, vascular smooth muscle cell, immune cells, enterocyte) in relation to lipid and immune metabolism, and heart function. CM = cardiomyocyte; EC = endothelial cell; VSMC = vascular smooth muscle cell; Th17—T helper 17 cells; Treg = Regulatory T cells; FXR = farnesoid X receptor; VDR = vitamin D receptor; PXR = pregnane R receptor; NR4A1 = nuclear receptor 4A1; TGR5 = Takeda G protein-coupled receptor; MR = muscarinic receptor; S1RP2 = sphingosine 1-phosphate receptor 2; SR-A1 = Class A1 scavenger receptors; AT2R = angiotensin II receptor type 2; TLR2—toll-like receptor 2; NCX = Na^+^/Ca^2+^ exchange protein; BKCa = large conductance Ca^2+^-activated K^+^; ASBT = apical sodium-dependent bile acid transporter; BA = bile acid; UDCA = ursodeoxycholic acid; (T/G)DCA = (tauro/glycol)deoxycholic acid; (T)LCA = Taurolithocholic acid; TCA = Taurocholic acid; CDCA = chenodeoxycholic acid; NF-κB = nuclear factor kappa B; PTP = Mitochondrial permeability transition pore; PLTP = phospholipid transfer protein; ET = endothelin-1; NO = nitric oxide; ROS = reactive oxygen species; PPARα = peroxisome proliferator-activated receptor alpha; SREBP1c = sterol regulatory element binding protein 1c; CYP7A1 = cytochrome P450 7A1; CYP39A1 = cytochrome P450 39A1; ApoA-IV = Apolipoprotein A-IV; NLRP3/4 = NLR family pyrin; FOXP3 = forkhead box P3; SMPD3 = Sphingomyelin Phosphodiesterase 3; SERCA2B = Sarcoendoplasmic reticulum calcium ATPase 2b; FGF15/19 = fibroblast growth factor 15/19; Ang1 = angiopoietin 1; iNOS = nitric oxide synthase; IL = interleukin; TJ = tight junction; * = hydrophobic bile acid pool; # = hydrophilic bile acid pool. This illustration was created with Biorender.com.

In this section, the focus switches from association to causality regarding the potential roles of BAs in CVD. The multifaceted roles of BAs in lipid homeostasis, immunity, and heart function indicate the ability to mediate CVD, as discussed in the following sections (Figure 2).

### 6.1. Regulation of Lipid Metabolism

Recent studies have shown that the elevation of plasma triglycerides (TG) comprises the major lipid abnormality in patients with atherosclerosis, changing the focus from LDL cholesterol to TG as being causal in atherosclerosis [2]. Interestingly, BAs appear to display beneficial effects in hypertriglyceridemia [95]. BA activation of the FXR-SHP pathway interferes with the regulation of fatty acid biosynthesis genes, mediated by the liver X receptor (LXR) and sterol regulatory element binding protein 1c (SREBP-1c) [95]. Feeding mice for 8 weeks with 0.5% CA was associated with a reduction in hepatic SREBP-1c expression and its lipogenic target genes in mouse models [95]. It has been proposed that the rate of hepatic lipogenesis is a major determinant of very-low-density lipoprotein (VLDL)-TG production, although it has been reported that massive lipogenesis in obese ob/ob mice does not lead to increased VLDL production [96]. While SREBP controls fatty acid synthesis, peroxisome proliferator-activated receptor α (PPARα) promotes fatty acid β-oxidation [97,98,99]. Th activation of PPARα by fenofibrate reduced the plasma TG’s, adiposity, and atherosclerosis development in high-fat diet-fed Ldlr^−/−^ mice [100,101]. Lipid accumulation in the aorta was prevented upon PPARα activation, probably by enhanced fatty oxidation. BAs can also induce PPARα via the activation of FXR [102].

In contrast with these beneficial effects in hypertriglyceridemia, BA activation of PXR, a well-known transcription regulator in the control of lipid metabolism [50,103], increased levels of total cholesterol, VLDL, and LDL; decreased high-density lipoprotein (HDL) levels; and increased atherosclerosis in ApoE^−/−^ mice [104], while PXR deletion reduced atherosclerosis in ApoE^−/−^ mice [105]. The genes involved in lipoprotein transport and cholesterol metabolism, including apolipoprotein A-IV (ApoA-IV), cytochrome P450 family 39 subfamily A member 1 (Cyp39a1), and cluster of differentiation 36 (Cd36), were affected upon PXR activation [104]. ApoA-IV and Cyp39a1 were down-regulated, whereas Cd36 was upregulated. Studies have indicated that CD36 plays an important role in atherosclerosis by mediating oxidized LDL (oxLDL) uptake by macrophages, leading to the formation of foam cells [106]. PXR activation in peritoneal macrophages led to increased Cd36 expression, which was consistent with increased lipid accumulation in these cells [104]. In addition, GUDCA was shown to inhibit macrophage foam cell formation by down-regulating scavenger receptor A1 gene expression, whereas Cd36 expression was not affected, implicating a different mode of action [82].

Of note, compensatory mechanisms preventing lipid accumulation in the circulation have been observed while directly targeting BA synthesis in CYP27A1/ApoE double-knockout (DKO) mice [50]. Zurkinden et al. showed that loss of the BA synthesis gene Cyp27a1 in ApoE^−/−^ mice fed a Western-type diet (WD) was associated with an upregulation of Cyp7a1 and cytochrome P450 3A1 (Cyp3a1), resulting in increased BA synthesis and excretion (i.e., accelerated cholesterol turnover), and protection against atherosclerosis [107]. In this study, they also observed differential cardiovascular outcomes of CA and CDCA feeding, in addition to the WD. Despite the observation that both feeding regimes led to reduced Cyp7a1 and Cyp3a1 expression, only CA-WD resulted in a strong increase in atherosclerosis, together with increased LDL and reduced HDL in the serum, increased intestinal absorption of cholesterol, and decreased faecal cholesterol output [107]. Although in humans CDCA is a more potent FXR agonist than CA, in rodents, FXR is mostly activated by CA as CDCA is rapidly converted in MCAs (FXR antagonists) [108]. Macrophage FXR activation by CA takes part in reverse cholesterol transport and reduces the HDL efflux [107]. In addition, the hydrophilic MCAs in the BA pool in CDCA-WD fed mice are likely responsible for the reduced cholesterol absorption [107], as the 12α-OH group of C7 appears to be essential for efficient cholesterol uptake by enterocytes [109].

Similar to BA sequestration, directly blocking intestinal BA absorption also affects BA synthesis. Targeting ASBT leads to the increased expression of BA synthesis genes and HMG-CoA reductase in ApoE^−/−^ mice [110]. To fuel the liver with additional cholesterol, hepatocytes increase both the de novo synthesis and expression of the cell surface LDL receptors, which results in reduced plasma cholesterol and less prominent aortic lesions.

FXR and TGR5 are both expressed in the vasculature. Whereas FXR is found in vascular smooth muscle cells (VSMCs) and endothelial cells of the vascular wall [111] (and possibly in macrophages [112]), TGR5 is expressed on the surface of macrophages [113], which are present in atherosclerotic plaque. Studies have shown that the activation of FXR in rat and human VSMCs reduces migration and inflammation [114], and affects lipid metabolism [111]; the latter particularly via phospholipid transfer protein (PLTP), an important regulator of reverse cholesterol transport. Moreover, FXR activation has been shown to mediate vasodilation in endothelial cells [115] and TGR5 activation to reduce inflammatory responses in macrophages [116].

Studies have evaluated the effect of TGR5 and/or FXR activation on atherogenesis [116,117]. The activation of TGR5 by INT-777 attenuated atherosclerosis development in Ldlr^−/−^ mice. INT-777 treatment led to reduced macrophage lipid loading and pro-inflammatory cytokine production, an effect mediated by altered cAMP-signalling and nuclear factor-κB (NF-kB) inhibition [116]. In the absence of TGR5, these anti-atherosclerotic effects were abolished [116]. Similarly, simultaneous activation of FXR and TGR5 by the dual agonist INT-767 in Ldlr^−/−^ mice reduced atherosclerosis via anti-inflammatory and lipid-lowering effects [117]. FXR deficiency alone completely blocked the lipid-lowering effects of INT-767: this was not found in TGR5-deficient mice. Interestingly, both FXR- and TGR5-deficient mice show reduced atherosclerosis and aortic inflammation upon INT-767 administration [117].

Thus, the anti-atherosclerotic effect of FXR and TGR5 activation is driven by inflammation, and the loss of one receptor is compensated by the other. Moreover, these anti-atherosclerotic effects of INT-767 were abolished by the dual deficiency of FXR and TGR5 in Ldlr^−/−^ mice [117]. In addition, Jadhav et al. found that INT-767 induced thermogenesis genes and reduced hepatic de novo lipogenesis genes, suggesting an additional role in energy homeostasis on top of the anti-atherosclerotic effects of this compound [118].

Intriguingly, both the deficiency and activation of FXR leads to similar results in atherosclerosis mouse models. FXR loss of function results in a decreased atherosclerotic plaque surface in Ldlr^−/−^ and ApoE^−/−^ mice [119,120]. Although the serum cholesterol levels were reduced, the TGs were elevated in these models. Peritoneal macrophages showed a reduced Cd36 gene expression and decreased lipid accumulation in FXR-LDLR DKO mice [120]. This suggests an indirect effect of FXR on macrophages. FXR activation also showed an atheroprotective effect in atherosclerosis models, which was in part the result of an improved lipid profile [117,118,121,122]. TG-lowering effects can be controlled by FXR-induced lipoprotein lipase and SREPB-1c activity [121]. Other anti-atherosclerotic effects could be the result of increased fecal cholesterol excretion and macrophage reverse cholesterol transport due to reduced BA pool size and composition [122]. Thus, both loss of function and activation of FXR attenuates atherogenesis in mice, which complicates the interpretation of these results.

Recent research has demonstrated that patients with hypercholesterolemia have elevated fibroblast growth factor 19 (FGF19), which is positively correlated with pro-atherogenic ceramide levels [123]. Intestinal FXR activation releases FGF19 (or fibroblast growth factor 15 (FGF15) in rodents) in the ileum, which is a negative regulator of hepatic BA synthesis. Intestinal FXR-deficient ApoE^−/−^ mice showed decreased atherosclerotic lesions in the aorta and heart, and reduced serum levels of ceramides after high-cholesterol diet feeding [123]. The enzyme sphingomyelin phosphodiesterase 3 (SMPD3) was identified as an intestinal FXR target, which is involved in ceramide synthesis. The overexpression of SMPD3 eliminated the anti-atherosclerotic effects in intestinal FXR-deficient ApoE^−/−^ mice [123].

Thus, BAs play an important role in lipid metabolism, as BAs affect BA and lipid synthesis, absorption, and excretion, but are also involved in foam cell formation and ceramide synthesis, which are both associated with CVD. Although it is not always clear whether the lipid-lowering (anti-atherosclerotic) effects of BAs are caused by direct or indirect effects of BA signalling, BAs represent an interesting therapeutic target for CVD.

### 6.2. Regulation of Immune Functions by Secondary Bile Acids

Inflammation plays a central role in CVD, involving both the innate and adaptive immune systems [12,124,125,126,127,128,129,130]. Alterations in the gut microbiota are associated with impaired gut integrity, leading to increased leakage of microbiota-derived LPS, which promotes systemic inflammation [12]. Targeting innate immunity with antibodies against interleukin-1 beta (IL-1β) led to reduced recurrent cardiovascular events in patients with previous myocardial infarction, independent of lipid lowering [131], suggesting that CVD is driven by inflammation. Interestingly, among the continuously growing list of metabolites regulating the immune system, secondary BAs are recognized to possess immuno-regulatory properties that affect CVD development [132].

FXR signalling is evidently involved in the maintenance of intestinal integrity and the regulation of inflammatory processes. For example, intestinal FXR activation has been shown to protect mice from intestinal inflammation through the downregulation of key pro-inflammatory cytokines [133], which are known to increase the permeability of the intestinal epithelial monolayer [133,134]. A different study showed that the genes encoding tight junction proteins, zonulin-1 and occludin, were increased upon FXR activation in a cirrhotic rat model [135]. In addition, FXR has been shown to induce the expression of genes such as angiopoietin 1 (Ang1), inducible NO synthase (iNOS), and interleukin-18 (Il-18), which show anti-microbial effects and protect the mucosal intestinal barrier in mice [136]. VDR is also involved in maintaining gut integrity and can be activated by LCA and iso-LCA [34,137,138]. The activation of VDR by LCA showed a protective effect on tumor necrosis factor alpha (TNFα)-induced injury of intestinal barrier function in Caco-2 cells, possibly through the NFκB signalling pathway [137]. Moreover, cell experiments demonstrated that LCA can induce adhesion molecule expression in endothelial cells through the activation of reactive oxygen species formation, NF-κB, and p38 mitogen-activated protein kinase (MAPK) signalling [139]. Increased surface adhesion molecules can attract immune cells to the vascular wall. Controversially, studies have shown anti-inflammatory effects upon VDR activation in CVD [50,140,141,142].

On the contrary, in vivo and in vitro studies have shown that the prolonged presence of excess DCA can reduce gut barrier function and promote intestinal inflammation [143]. DCA may serve as an endogenous danger-associated molecular pattern (DAMP) and activate inflammasome NOD-, LRR-, and pyrin domain-containing protein 3 (NLRP3), a cytosolic multiprotein of the innate immune system, which promotes the secretion of pro-inflammatory cytokines [144]. DCA binds to the sphingosine-1-phosphate receptor 2 (S1PR2) in macrophages that mediates NLRP3 activation [144]. Moreover, Wang et al. found that HFD-induced dysbiosis promotes DCA production, leading to increased pro-inflammatory colonic macrophage infiltration in mice [145]. DCA dose-dependently promoted M1 macrophage polarization and cytokine production via the NF-kB signalling pathway, partially through toll-like receptor 2 (TLR2) and muscarinic 2 receptor (M_2_R) activation.

TGR5 activation in CD4 T cells inhibits inflammation by regulating the recruitment of CD4 and CD8 T cells after myocardial infarction [146]. In line with this anti-inflammatory effect, the administration of TLCA led to NLRP3 inflammasome inhibition via the TGR5-cAMP-PKA axis in T cells and macrophages. PKA can induce the phosphorylation of NLRP3, thereby preventing the activation of the inflammasome [147]. Interestingly, SHP is a negative regulator of NLPR3 inflammasome activation and its deficiency showed increased pro-inflammatory cytokines in vivo, including Il-1β and Il-18 [148].

Similar to FXR and TGR5, VDR activation exhibits strong anti-inflammatory effects in macrophages [149,150,151]. VDR deletion increased foam cell formation from a lack of the VDR-sarcoendoplasmic reticulum calcium ATPase 2b (SERCA2b) interaction, causing activation of ER stress and the induction of CD36 and class A1 scavenger receptor (SR-A1) in the macrophages [149]. SERCA2b, is a critical enzyme that maintains ER calcium levels to optimize protein production and folding [149].

Accumulating evidence indicates that an altered microbiota can activate both the innate and adaptive immune system [83,84,85]. Western-type diet-induced dysbiosis [83] and microbes from CAD patients [84] was shown to modulate BA pool composition, worsen gut barrier permeability, and increase systemic and intestinal inflammation, likely by imbalanced T helper and T regulatory cells (Tregs) in the intestinal lamina propria. A higher ratio of retinoid-acid-related orphan receptor-γt (RORγt) T helper 17 cells to (Helios+) regulatory T cells was observed [83,84]. While transcription factor RORγt is required for the differentiation of pro-inflammatory Th17 cells, orchestrating intestinal inflammatory responses, Helios+ cells play a critical role in tissue repair, and in the maintenance of gut barrier function [152]. Chen et al. speculated that BAs and other oxysterols can serve as endogenous RORγt agonists, modulating different T cell subsets [83]. Indeed, fecal LCA is positively correlated with the Th17−Treg ratio in the intestinal lamina propria [84], suggesting that signals from the gut microbiota could drive the differentiation and maintenance of T cell subsets in the intestine. In addition, microbes from CAD patients led to increased reactive oxygen species generation and vascular stiffness in the aorta, probably caused by increased intestinal inflammation and worsened gut barrier permeability [78].

Follow-up studies have shown that derivates from LCA can modulate T cell subsets. While 3-oxoLCA inhibited the differentiation of Th17 through direct binding to RORγt [138,153], iso-alloLCA increased the differentiation of Tregs [138] in a nuclear hormone receptor Nur77-dependent manner [154], enhancing Forkhead Box P3 (FOXP3), a master regulator in the development and functioning of Tregs. Changing the BA pool by manipulating dietary and microbial factors confirmed the role of BAs in modulating FOXP3 Tregs in the colon [155]. The genetic ablation of BA enzymes in the bacteria reduced Tregs, which was resolved after restoring the BA content in the intestine. Moreover, a nutrient minimal diet lowered fecal deconjugated BAs and reduced Tregs in the colon, which was only restored by the administration of LCA/3-oxo-LCA [155]. Investigating different BA receptors in modulating colonic Treg cell populations demonstrated a major role of the BA-VDR pathway [155]. VDR is expressed in FOXP3 Tregs, but also in endothelial and dendritic cells in the colon [155]. The secondary BA isoDCA can increase FOXP3 Treg induction by acting on dendritic cells [156]. Dendritic cell FXR ablation led to increased Treg production, showing the same transcriptional profile compared to isoDCA induction [156]. The role of VDR in this process was not investigated. Interestingly, human VDR genetic variants could affect intestinal inflammation through controlling the Treg pool [157]. Forward genetics studies have observed polymorphisms in the VDR promotor, which controlled VDR expression and T cell activation [157]. The different activation status of VDR could affect intestinal inflammatory susceptibility through the improper control of Tregs in the colon, driving low-grade systemic inflammation [157].

Thus, both the innate and adaptive immune systems can be modulated by BA signalling, either by boosting or inhibiting inflammatory responses. Therefore, (indirectly) targeting BA metabolism to reduce systemic inflammation could be a new therapeutic opportunity for CVD.

### 6.3. Regulation of Heart Function

Studies have shown a direct role of BAs in features of heart and arteries (cardiovascular tissue). BAs appear to regulate cardiovascular function through the activation of BARs (FXR, TGR5, and VDR) and MRs, as well as through the interaction with ion channels in cardiovascular tissue [31]. This section summarizes BA signalling in relation to cardiovascular function.

FXR is highly expressed in the liver, kidney, and gastrointestinal tract, but is also expressed in heart tissue (i.e., cardiomyocytes and fibroblasts) and in the vasculature (i.e., endothelial and vascular smooth muscle cells) [111]. FXR activation results in cell-type specific responses [111,115,158,159,160,161]. FXR activation of isolated neonatal rat cardiomyocytes, either by natural (CDCA) or synthetic (GW4064) FXR activators, induced apoptosis [158]. Interestingly, increased Fxr expression was observed after myocardial ischaemia/reperfusion in mouse hearts, whereas both pharmacological inhibition or the genetic deletion of FXR reduced myocardial apoptosis, decreased infarct size, and improved the cardiac function of ischaemic hearts [158]. Hydrophilic UDCA was shown to protect the myocardium against reperfusion injury in rat hearts by blocking the opening of the cardiac mitochondrial permeability transition pore (PTP) during reperfusion of the heart [162].

Studies have also indicated a role of BAs in regulating vascular tension [115,161,163]. For example, activation of endothelial FXR led to downregulation of Endothelin-1 (Et-1) and Il-1 mRNA expression, both potent vasoconstrictive agents [115]. Additionally, FXR activation resulted in upregulation of endothelial nitric oxide synthase (eNOS) [161]. eNOS-derived nitric oxide (NO) has vasodilatory effects and plays an important role in vasomotor tone, VSMC proliferation, platelet aggregation, and the inhibition of lipid oxidation [164]. Mechanistic studies reported an FXR-responsive element in the eNOS promotor [161]. In vitro and in vivo studies demonstrated that, next to FXR, the activation of TGR5 also increased NO production in aortic endothelial cells, and reduced monocyte adhesion and the activation of NF-kB [163]. In addition, DCA treatment improved cardiac function by inhibiting Il-1β expression in the infarcted hearts of mice in cardiomyocytes via the TGR5-NFκB pathway [165]. In line with these results, serum DCA was reduced in acute myocardial patients [165].

FXR activation in VSMCs stimulates the angiotensin system [159,160]. FXR activation in rat aortic smooth muscle cells led to increased expression of angiotensin (Ang) II type 2 receptor (AT2R) and the inhibition of Ang II-mediated extracellular signal-regulated kinase (ERK) activation and cell proliferation [160]. Whereas angiotensin II Type I Receptor (AT1R) activation has vasopressor effects, AT2R activation has vasodilatory roles in the regulation of blood pressure [166]. These vasodilatory effects were impaired in cultured rabbit mesenteric arteries after chronic FXR activation. The FXR agonist GW4064 dose-dependently impaired endothelium relaxation, caused by the decreased sensibility of VSMCs to NO [159]. Thus, these studies suggest different roles in the regulation of blood pressure upon short- and long-term FXR activation.

Whole body FXR/SHP double knockout mice, a model of BA overload, displayed cardiac hypertrophy, bradycardia, and exercise intolerance [167]. Cardiac fatty acid oxidation was reduced in favour of glucose oxidation. Cholestatic mice also have elevated plasma BAs and show increased hypertrophic signalling in the heart, along with suppressed fatty acid oxidation and increased myocardial glycogen content [168]. Interestingly, reducing plasma BAs by intestinal BA sequestration reversed the observed heart dysfunction in FXR/SHP double knockout mice [167]. These results imply a role of serum BAs on heart function. Of note, mechanistic studies have demonstrated that only the deletion of SHP led to hypertrophy and bradycardia [167], suggesting that SHP is an important antihypertrophic regulator.

VDR signalling is also involved in regulating cardiac function [169,170]. Functional VDR is found in t-tubules of cardiac myocytes [169]. T-tubules regulate intracellular calcium flow and allow the heart to contract more forcefully [171]. Cardiac myocytes isolated from VDR knockout mice show increased rates of contraction, cardiac hypertrophy, and systolic and diastolic dysfunction compared with wild-type mice [169]. In addition, epidemiological studies have observed a link between vitamin D deficiency and CVD [172,173,174]. Vitamin D deficiency in mouse models leads to increased systolic and diastolic blood pressure, high plasma renin and decreased urinary sodium excretion, and increased atherosclerosis in the aortic arch accompanied by increased macrophage/foam cell infiltration with ER stress activation [150]. Interestingly, vitamin D supplementation improved left ventricular (LV) function and reversed LV remodelling in heart failure (HF) patients [170]. As LCA is a potent endogenous ligand of VDR in T cells, it may be speculated whether these specific BAs can also activate VDR in other cell-types, such as myocytes. If this is the case, LCA-VDR signalling in myocytes could be involved in the modulation of heart function.

BAs also bind to “non-classical BAR”, such as muscarinic (acetylcholine) receptors (MR) [31]. MRs are G-protein coupled receptors and are classified into different subtypes, namely M_1_R–M_5_R [31]. Molecular modelling shows strong similarities in the molecular surface of acetylcholine and BAs [175]. In contrast with acetylcholine, BA-specific MR activation depends on hydrophobic interactions. Hence, conjugates of (hydrophobic) secondary BAs showed favourable binding to M_3_R, acting as antagonists [175,176,177,178]. Tauro-LCA (TLCA) and conjugates of DCA are bound to M_3_R and inhibit acetylcholine-induced increases in inositol phosphate formation and MAPK phosphorylation [176,177]. Moreover, tauro-DCA (TDCA) stimulated vasodilatory actions in rat thoracic aortae rings, in part by an NO-, M_3_R-dependent mechanism [179]. Additionally, conjugated BAs were found to be partial agonists of M_2_R, slowing the contraction rate in neonatal mesenteric vascular muscle cells [180]. Tauro-CA (TCA) was found to interact with M_2_R on neonatal rat cardiomyocytes, lowering intracellular cAMP and inducing arrhythmia in cardiac tissue. Arrhythmia was caused by reduced myocardial cell contraction [181]. Importantly, reduced contraction could also be the consequence of cytotoxicity at higher BA concentrations [180]. The effects of BAs on the remaining MRs in the heart remain to be explored.

In addition to binding to BAR and MRs, BAs can interact with ion channels, such as the large conductance Ca^2+^-activated K^+^ (BKCa) channel [182,183] and Na^+^/Ca^2+^ exchange protein (NCX) [184]. Natural BAs and synthetic analogues show direct binding to BKCa channels, increasing their activity and leading to the relaxation of rabbit mesenteric artery smooth muscle cells [182]. For example, LCA was found to mediate BKCa channel activation causing relaxation in the small arteries [183]. Moreover, TCA dose-dependently induced arrhythmias in adult human atrial tissue, probably by depolarization of the resting membrane potential, enhancing the NCX current density, and inducing after polarizations [184]. These effects were prevented after NCX inhibition [184].

To summarize, BAs are recognized signalling molecules in modulating cardiovascular function. Their action is mediated by BARs, MRs, or ion channel interaction and mostly leads to vasodilatory effects. The hydrophobicity, polarity, and/or conjugation state of BAs seems to play an important role in the magnitude of receptor/channel activation. Future studies should uncover BA effects in cardiac tissue to exploit BA-mediated targets in order to control heart disease.

## 7. Bile-Acid-Based Therapies in Cardiovascular Disease

As summarized above, BAs act as important hormonal signalling molecules that modulate cardiovascular function. Given the new insights of BAs in lipid metabolism, immunity, and heart function, strategies for BA-based treatment of CVD can be considered. Targeting BA metabolism could either be indirectly (via gut microbiota) or directly (BAR modulators). In this section, we briefly discuss potential BA-based therapies for the treatment of CVD.

### 7.1. Indirect Bile-Acid-Based Therapies

Targeting the gut microbiota (and thereby indirectly BA metabolism) can be accomplished via restoration (fecal microbiota transplantation (FMT)) of the gut flora. However, this strategy is unlikely to be suitable for future applications, given that the efficiency of FMT is still limited and inconclusive (due to non-specificity and engraftments problems) and there is risk of pathogenic infection [60]. Other approaches include prebiotics, probiotics, and synbiotics (mixture of pre and probiotics).

Prebiotics do not contain bacteria, but are substrates that are selectively utilized by host microorganisms, and thereby stimulate their growth [60]. Prebiotics (e.g., fibers and oligosaccharides) can be obtained from various sources, including raw oats, soybeans, and several plants [185]. Interestingly, dietary fibers can also regulate BA levels in the gut lumen by binding to conjugated BAs, and then serve as a platform for gut bacteria that possess BA-metabolizing enzymes [186]. A recent clinical trial demonstrated the health benefit of pea fiber in weight control and blood glucose levels [187], two important factors in managing cardiovascular function. Pea fiber can modulate the gut microbiota and alter fecal SCFAs and BAs [188]. For example, the genus *Oscillospira* was reduced after pea fiber treatment, which was negatively correlated with reduced abundances of BAs, such as DCA and isoLCA [188]. This study also reported an overall decrease in fecal BAs, including CA, CDCA, and DCA. This study indicates the potential role of prebiotics in modulating the microbiota and its metabolites, which contributes to metabolic health benefits in overweight patients (who are at risk for CVD) [187,188].

Probiotics are live bacteria with the entire molecular machinery of living cells, which have been shown various health benefits when administered in adequate amounts [60]. Preclinical and clinical studies have shown anti-atherogenic effects of probiotics (reviewed extensively in [189]). Probiotics (e.g., species of *Lactobaccillus* and *Bifidobacterium*) often contain a BSH activity, which is related to cholesterol-lowering effects in plasma [190]. Some probiotics can also increase BA synthesis via direct inhibitory actions on FXR and SHP [189,191]. In addition, probiotics seem to be associated with anti-inflammatory and anti-oxidative effects and enhanced endothelial function in arteries [192]. Besides the genera *Lactobacillus* and *Bifidobacterium*, lactic acid bacteria has been shown to have both fermentation productivity and antioxidant properties [193]. Moreover, as previously described, derivates from LCA can modulate T cell subsets (Figure 2)—3-oxoLCA and isoLCA inhibit the differentiation of Th17 cells [138,153], while iso-alloLCA increases the differentiation of Tregs [138]. Higher intestinal levels of these secondary BAs may be beneficial in controlling gut immune homeostasis and reducing systemic inflammation [155]. Screening bacteria in human stool samples for their ability to convert LCA into its derivates (3-oxo-, iso-, and iso-allo-LCA) using UPLC-MC traces, is a useful tool to select bacterial species as potential probiotics [153]. Paik et al. revealed that species *Eggerthella lenta* and *Ruminococcus gnavus* are able to convert LCA to 3-oxoLCA and isoLCA, while species *Bacteroides fragilis* convert 3-oxoLCA to isoLCA. The colonization of these species reduced Th17 cell levels in the colonic lamina propria in mice [153]. This study showed promising results of using probiotics to alter BA metabolism and control immune homeostasis in the gut [153]. Whether these effects could also influence heart function or atherosclerotic progression needs to be further investigated. Of note, probiotics do not always show beneficial effects, partly due to heterogeneity in the human population or engraftment problems [60].

To improve colonization, probiotics can be given as a mixture with prebiotics to promote survival and activity of these specific bacterial strains [60]. For example, administration of *Bifidobacterium animalis substrate Lactis 420* together with prebiotic Litesse Ultra polydextrose (consisting of fibers) show synergistic clinical effects on body fat regulation [194]. Synbiotic treatment also led to alterations in gut microbiota composition, BA composition (reduced conjugated BAs), improved gut barrier function, glucose tolerance, and mitigation of inflammation [194]. In addition, evidence from systematic reviews and meta-analysis of clinical trials lend support for synbiotic treatments to reduce plasma LDL and triglyceride levels and increase HDL, probably through reduced intestinal cholesterol absorption and higher BA excretion [195,196]. The effects of synbiotics were more effective when consumed for longer than 8 weeks [196].

### 7.2. Direct Bile-Acid-Based Therapies

As previously discussed, preclinical studies have shown lipid-lowering and anti-inflammatory effects for FXR and TGR5 agonists. These benefits make the receptors potential candidates for the treatment of CVD. The development of FXR and TGR5 modulators are in its early phase and clinical studies in patients with CVD are lacking. However, obeticholic acid (OCA), which is the first developed small molecule targeting FXR, was evaluated in clinical trials and showed promising results in patients with metabolic diseases [197]. OCA treatment (6 weeks) was well-tolerated and increased insulin sensitivity and reduced markers of liver inflammation and fibrosis in diabetic and non-alcoholic fatty liver disease (NAFLD) patients. As expected, a side effect of OCA treatment is increased serum cholesterol levels due to FXR activation and reduced hepatic CYP7A1 expression [197]. Evaluation of the lipid profile modulation by OCA treatment is currently ongoing. In addition, TGR5 agonists are in development as therapeutics for cardiometabolic diseases. For example, derivatives of CDCA have been tested to treat obesity, insulin sensitivity, and inflammation [198,199]. However, most TGR5 agonists are still in preclinical phase or showed unsatisfactory results in phase I trials [199]. Whether FXR and/or TGR5 agonists are interesting targets as treatment for CVD still needs to be determined.

Of note, mouse models have been widely used in preclinical studies to investigate the mechanistic roles of BAs in CVD. However, the marked species difference in BA metabolism between humans and mice have hampered the interpretation of the results [200,201,202]. As mentioned before, mice produce rodent-specific MCAs, which account for 35% of the total BA pool in mice [203]. Because of the hydrophilicity of MCAs, the murine BA pool is more hydrophilic than the human BA pool [203]. As BA species have dissimilar affinities for the activation of BARs, differences in BA composition could differentially affect lipid metabolism, immunity, and heart function. The depletion of the rodent-specific enzyme Cyp2c70, which is responsible for the production of MCAs, has been developed to humanize the BA pool composition in mice and is of great value in this respect [200,201,202]. In addition, the mouse specific enzyme Cyp2a12 was recently discovered [201]. This enzyme rehydroxylates DCA and LCA upon their arrival in the liver, giving rise to CA and MCAs. The proportion of secondary BAs are thus reduced in the pool in mice. Generation of Cyp2a12 knockout mice show the accumulation of DCA in the pool, resembling a more human-like BA pool [201]. These novel humanized BA mouse models will become of critical importance to bridge the gap between laboratory and clinical application, while preserving the benefits of the mouse as a preclinical model.

In addition, ex vivo models are being developed and validated to study atherosclerosis based on the actual human plaque (obtained from endarterectomy surgery) [204,205,206]. Although the complexity of the disease underscores the necessity to use in vivo (rodent) models to investigate the mechanisms of atherosclerosis, this ex vivo human atherosclerosis model displays major advantages and opportunities. Previous studies have shown that segments of the human plaque can be maintained for 2 weeks in culture [206], providing an attractive ex vivo model to acquire fundamental knowledge and study the impact of novel treatment strategies, such as FXR and TGR5 modulators, to provide a basis for future innovative therapeutics.

## 8. Conclusions

Evidence is accumulating that BAs exert a much broader range of biological functions than initially recognized, playing a part in mediating lipid metabolism, immunity, and heart function. Given the fact that human studies have shown disturbances in the gut microbiota and BA metabolism in relation to CVD, there are speculations that BA metabolism could be a potential therapeutic target in the future. However, our understanding of the mechanisms is mainly based on preclinical studies, and translational human studies are much needed. Nevertheless, BA-based therapeutics, either indirect or direct, show the potential to reduce the risk factors of CVD. Of note, due to the heterogeneity in the human population, a “one size fits all” approach is expected to not be successful (especially when targeting the gut microbiota). In conclusion, BAs are important signalling molecules in the human body, acting as integrators and modulators of important cardiometabolic pathways. A better and more comprehensive understanding of BAs in cardiovascular responses will be of great importance in the establishment of novel therapeutic approaches to combat CVD.

## Figures and Tables

**Table 1:** Cross-sectional studies on the gut microbiota composition in atherosclerotic patients.

Author	Population	Atherosclerosis Definition	Sequencing Method	Higher Abundance in Atherosclerosis	Lower Abundance in Atherosclerosis	Microbial Diversity in Atherosclerosis	Covariates in Analyses
Zheng et al., 2020 [63]	152 patients 105 controls	Atherosclerosis (≥50% stenosis in one or more vessels)	16S	*Bulleidia*, *Comamonas*, *Enhydrobacter*	*Agrobacterium*, *Delftia*, *Enterobacter*, *Morganella*	Increased	Unadjusted
Karlsson et al., 2012 [64]	12 patients 13 controls	Symptomatic atherosclerosis (who had undergone CAE)	Shotgun	*Collinsella* (7α-HSDH BSH-T4)	*Roseburia* (BSH-T1), *Eubacterium* (7α-HSDH/BSH-T1)	NR	Smoking, diabetes, age and BMI
Zhu et al., 2018 [65]	70 patients 98 controls	Atherosclerosis (confirmed by coronary angiography)	16S	*Escherichia-Shigella*, Enterococcus (BSH)	*Roseburia* (BSH-T1), *Eubacterium* rectale (7α-HSDH/BSH-T1), *Faecalibacterium*, *Enterococcus* (BSH-T0)	Decreased	Unadjusted
Yin et al., 2015 [66]	141 patients 94 controls	Symptomatic atherosclerosis (with TIA)	16S	*Enterobacter*, *Megaspaera*, *Desulfovibrio*	*Bacteroides* (7α-HSDH/BSH-T5/6), *Prevotella*, *Faecalibacterium*	Increased	Unadjusted
Jie et al., 2017 [67]	218 patients 187 controsl	Atherosclerosis (≥50% stenosis in one or more vessels)	Shotgun	*Enterobacteriaceae*, *Streptococcus* spp. (BSH-T2)	*Roseburia* (BSH-T1), *Faecalibacterium Prausnitzii*	No difference	Unadjusted
Toya et al., 2020 [68]	53 patients 53 controls	Atherosclerosis (≥50% stenosis in one or more vessels)	16S	*Ruminococcus gnavus* (3α-HSDH BSH-T1)	*Lachnospiraceae NK4B4*, *Ruminococcus Gauvreauii* (BSH-T1)	Decreased	Age, sex, race, BMI, DM, dyslipidemia
Liu et al., 2019 [69]	161 patients 40 controls	Atherosclerosis (≥50% stenosis in one or more vessels)	16S	*Veillonella*, *Haemophilus*, *Klebsiella*	*Roseburia* (BSH-T1), *Faecalibacterium*	No difference	Unadjusted
Feng et al., 2016 [70]	59 patients 43 controls	Atherosclerosis (confirmed by coronary angiography)	Shotgun	*Clostridium* sp. *HGF2* (BSH-T0), *Streptococcus* sp. *M334/M143* (BSH-T2)	NR	NR	Unadjusted
Yoshida et al., 2018 [71]	30 patients 30 controls with risk factors	Atherosclerosis (≥75% stenosis in one or more vessels) AND stable angina pectoris, MI	16S	*Faecalibacterium prausnitzii*, *Prevotella copri*	*Bacteroides vulgatus*, *Bacteroides dorei* (BSH-T5/6)	No difference	Age, sex

## Data Availability

Not applicable.
